# Supplementary material for: Distribution of yeast species and their resistance to copper and sulfite across arboreal and viticulture habitats
Source: FEMS Yeast Res. 2025 Dec 17;26:foaf074. doi: 10.1093/femsyr/foaf074 (PMC12767201; doi:10.1093/femsyr/foaf074)
Supplement: foaf074_Supplemental_Files [file foaf074_supplemental_files.zip › Supplemental_Figures.pdf]

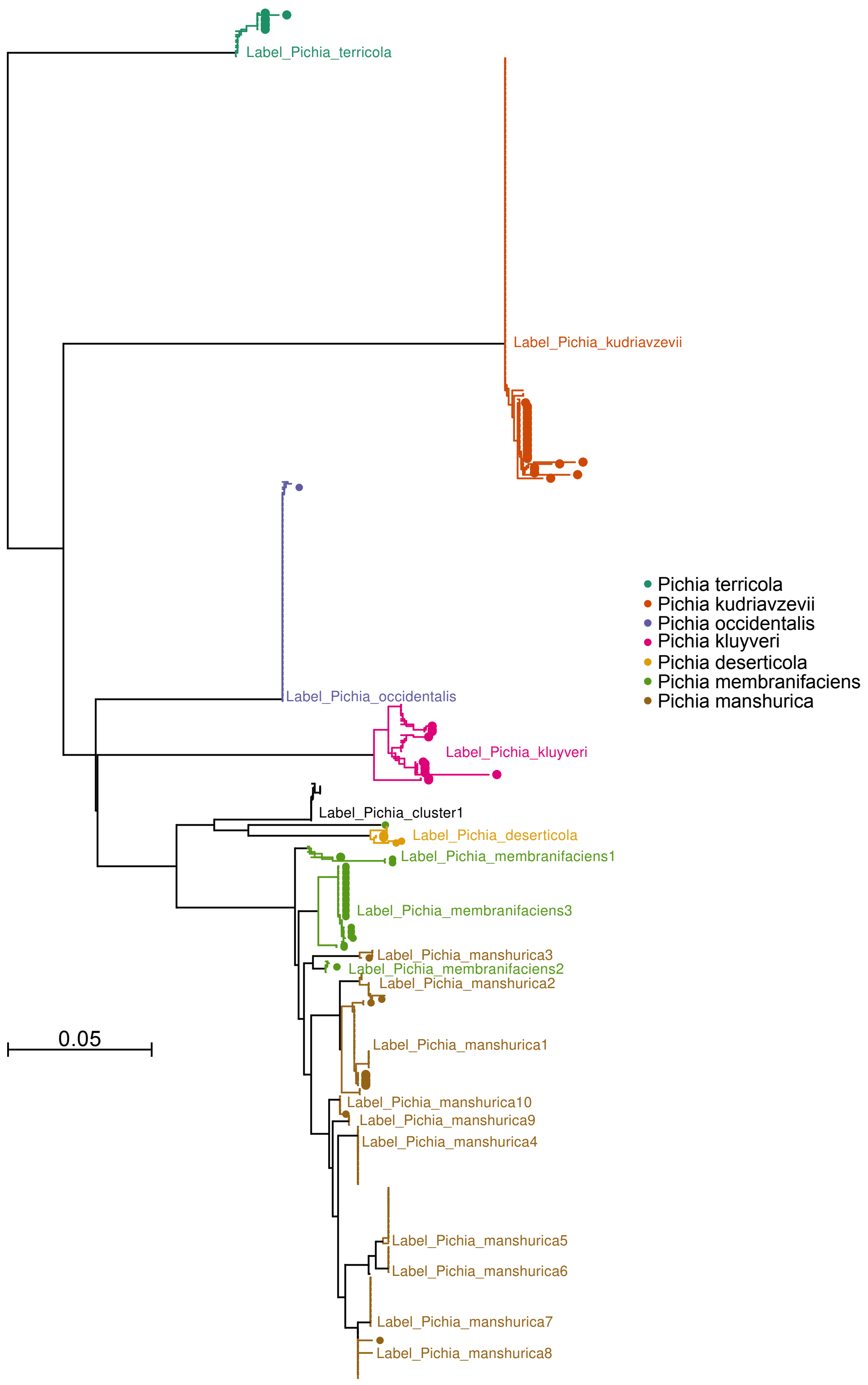

Figure S1. Phylogenetic relationship of Pichia species and sequenced strains. Phylogeny shows known species (circles) and strains (no label) based on ITS2 sequences. Strain branches are colored according to the species or group labels.

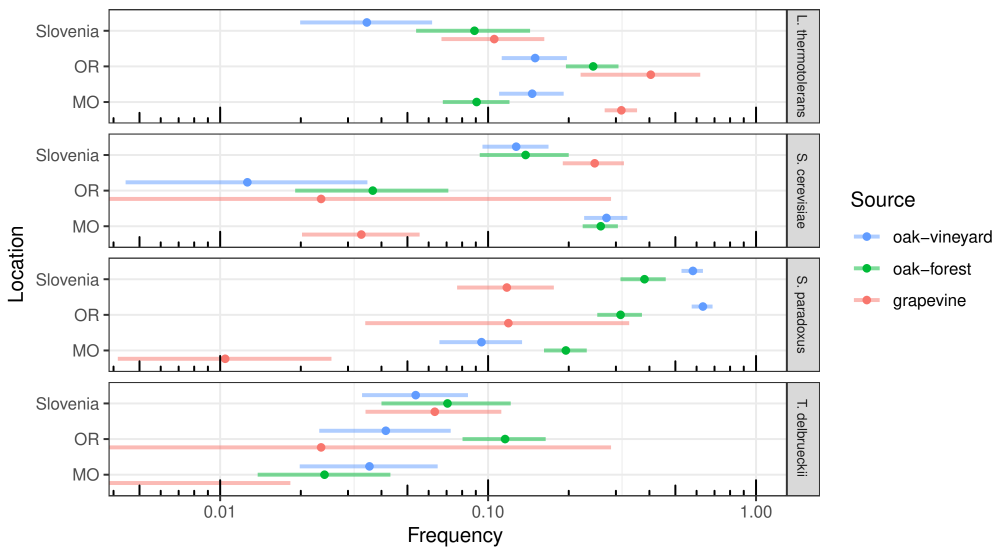

Figure S2. Species frequency across regions and sample sources. Each panel (species) shows species frequency in Missouri (MO), Oregon (OR) and Slovenia for samples of grapevines and vineyard and non-vineyard oaks. Bars show 95% confidence intervals.

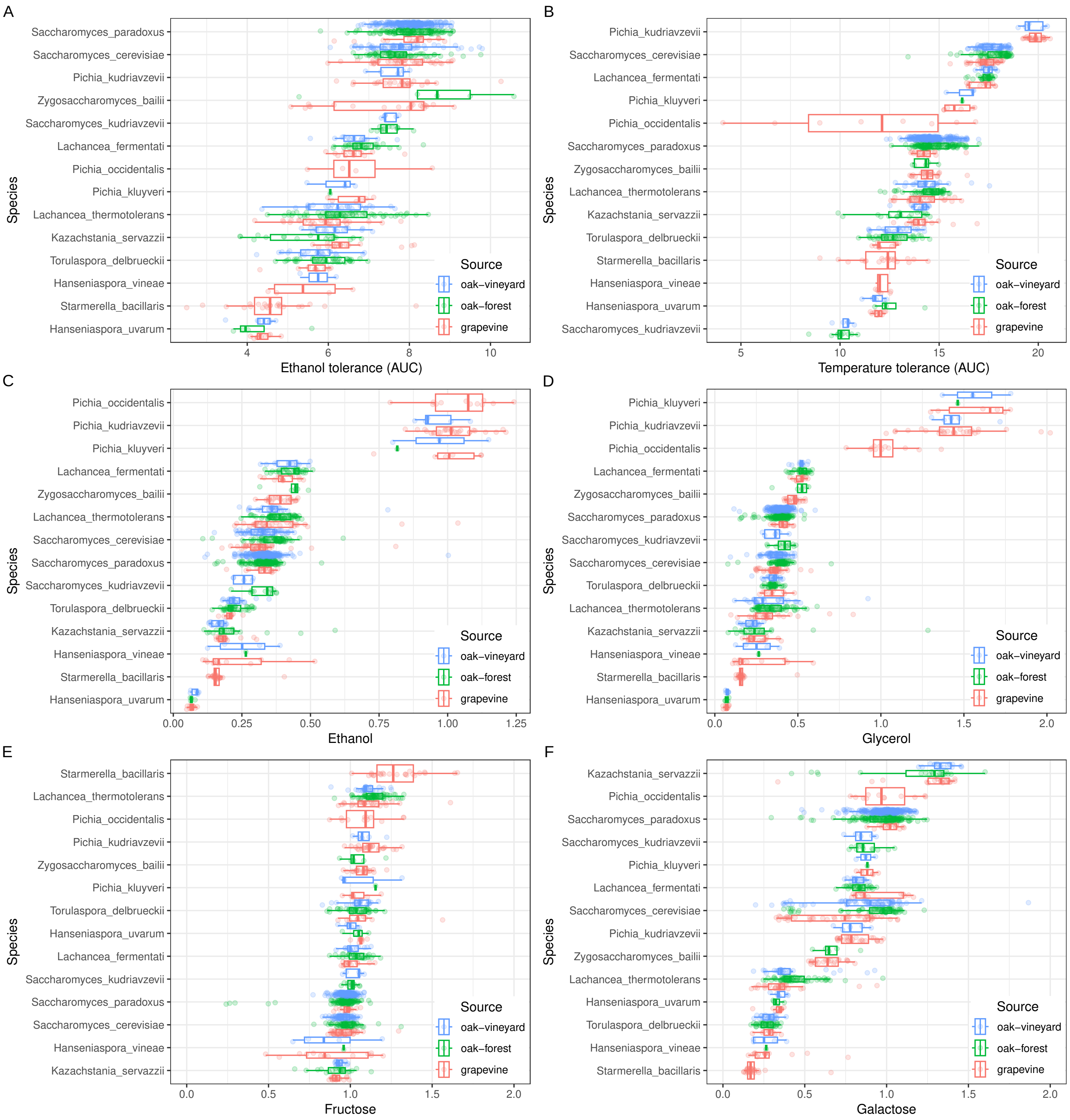
A
Saccharomyces_paradoxus
Saccharomyces_cerevisiae
Pichia_kudriavzevii
Zygosaccharomyces_bailii
Saccharomyces_kudriavzevii
Lachancea_fermentati
Pichia_occidentalis
Pichia_kluyveri
Lachancea_thermotolerans
Kazachstania_servazzii
Torulaspora_delbrueckii
Hanseniaspora_vineae
Starmerella_bacillaris
Hanseniaspora_uvarum
Species
4
6
8
10
Ethanol tolerance (AUC)
Source
oak−vineyard
oak−forest
grapevine
B
Pichia_kudriavzevii
Saccharomyces_cerevisiae
Lachancea_fermentati
Pichia_kluyveri
Pichia_occidentalis
Saccharomyces_paradoxus
Zygosaccharomyces_bailii
Lachancea_thermotolerans
Kazachstania_servazzii
Torulaspora_delbrueckii
Starmerella_bacillaris
Hanseniaspora_vineae
Hanseniaspora_uvarum
Saccharomyces_kudriavzevii
Species
5
10
15
20
Temperature tolerance (AUC)
Source
oak−vineyard
oak−forest
grapevine
C
Pichia_occidentalis
Pichia_kudriavzevii
Pichia_kluyveri
Lachancea_fermentati
Zygosaccharomyces_bailii
Lachancea_thermotolerans
Saccharomyces_cerevisiae
Saccharomyces_paradoxus
Saccharomyces_kudriavzevii
Torulaspora_delbrueckii
Kazachstania_servazzii
Hanseniaspora_vineae
Starmerella_bacillaris
Hanseniaspora_uvarum
Species
0.00
0.25
0.50
0.75
1.00
1.25
Ethanol
Source
oak−vineyard
oak−forest
grapevine
D
Pichia_kluyveri
Pichia_kudriavzevii
Pichia_occidentalis
Lachancea_fermentati
Zygosaccharomyces_bailii
Saccharomyces_paradoxus
Saccharomyces_kudriavzevii
Saccharomyces_cerevisiae
Torulaspora_delbrueckii
Lachancea_thermotolerans
Kazachstania_servazzii
Hanseniaspora_vineae
Starmerella_bacillaris
Hanseniaspora_uvarum
Species
0.0
0.5
1.0
1.5
2.0
Glycerol
Source
oak−vineyard
oak−forest
grapevine
E
Starmerella_bacillaris
Lachancea_thermotolerans
Pichia_occidentalis
Pichia_kudriavzevii
Zygosaccharomyces_bailii
Pichia_kluyveri
Torulaspora_delbrueckii
Hanseniaspora_uvarum
Lachancea_fermentati
Saccharomyces_kudriavzevii
Saccharomyces_paradoxus
Saccharomyces_cerevisiae
Hanseniaspora_vineae
Kazachstania_servazzii
Species
0.0
0.5
1.0
1.5
2.0
Fructose
Source
oak−vineyard
oak−forest
grapevine
F
Kazachstania_servazzii
Pichia_occidentalis
Saccharomyces_paradoxus
Saccharomyces_kudriavzevii
Pichia_kluyveri
Lachancea_fermentati
Saccharomyces_cerevisiae
Pichia_kudriavzevii
Zygosaccharomyces_bailii
Lachancea_thermotolerans
Hanseniaspora_uvarum
Torulaspora_delbrueckii
Hanseniaspora_vineae
Starmerella_bacillaris
Species
0.0
0.5
1.0
1.5
2.0
Galactose
Source
oak−vineyard
oak−forest
grapevine

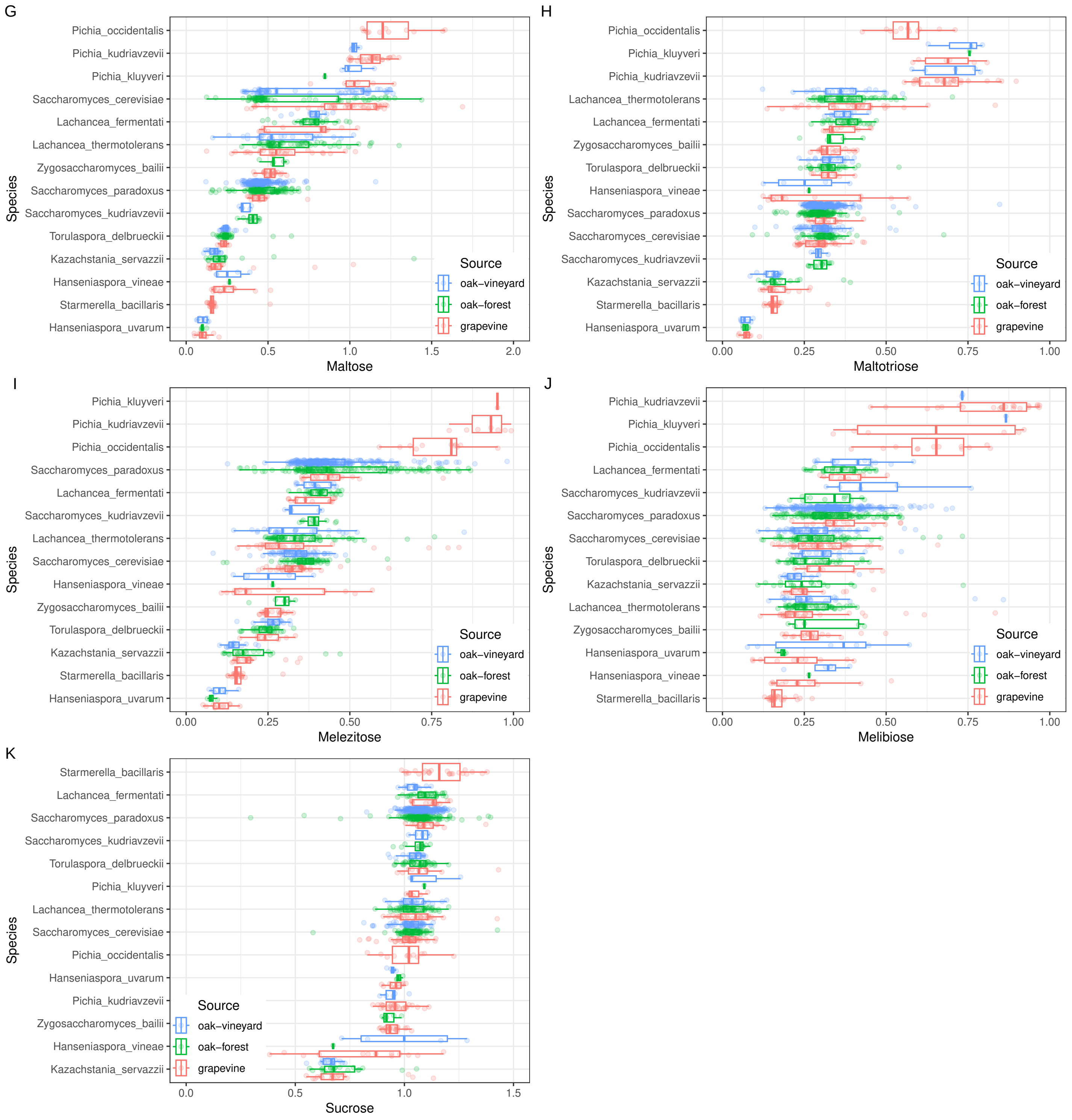

Figure S3. Phenotypic variation within and between species. Phenotypic variation is shown for 14 species with ten or more strains in the subset used for extended phenotyping. Ethanol (A) and temperature (B) tolerance was measured by area under the curve (AUC) normalized to no ethanol and 23 °C, respectively. On this scale AUC is in units of percent ethanol or degrees above 23. Growth on ethanol (C), glycerol (D), fructose (E), galactose (F), maltose (G), maltotriose (H), melezitose (I), melibiose (J) and sucrose (K) is colony size relative to growth on glucose. Strains (points) are colored according to whether they came from grapevine, oak-forest or oak-vineyard samples (source) and boxplots show median and interquartile range. Species were ordered with respect to mean normalized colony size.
